# Association of Nocturnal Enuresis With Vesicoureteral Reflux and Renal Cortical Damage

**DOI:** 10.5812/numonthly.2030

**Published:** 2012-03-01

**Authors:** Mitra Naseri

**Affiliations:** 1 Pediatric Nephrology Department, Dr Sheikh Children hospital, Mashhad University of Medical Sciences, Mashhad, IR Iran

**Keywords:** Nocturnal Enuresis, Diurnal Enuresis, Vesicoureteral Reflux

## Abstract

**Background:**

The prevalence of vesicoureteral reflux (VUR) is higher in enuretic children than in non-enuretic children. Recent studies have reported VUR in 6–23% of children with enuresis.

**Objectives:**

To clarify the association of nocturnal enuresis with vesicoureteral reflux (VUR) and to identify children who are at risk for VUR.

**Patients and Methods:**

During 2007–2009, neurologically normal children who were referred with a chief complaint of nocturnal enuresis and had abnormal renal ultrasonography (US) results, daytime incontinence, abnormal results in urodynamic studies, urinary tract infection, or a history of VUR in their siblings were prospectively evaluated for VUR by voiding cystourethrography (VCUG).

**Results:**

A total of 60 children (26 boys and 34 girls) aged 5–17 (mean ± SD: 8.46 ± 2.45) years met the inclusion criteria and were enrolled in the study. Twenty-eight (46.7%) patients had mono-symptomatic nocturnal enuresis (MNE), and 32 (53.3%) had non-mono symptomatic nocturnal enuresis (NMNE). VUR was reported in 10 (16.7%) patients and posterior urethral valve (PUV) was found in 1 (1.7%) patient. The prevalence of VUR was significantly higher in patients with daytime incontinence and in girls (P = 0.016 and 0.003 respectively). We did not find any significant correlations between VUR and the form of enuresis (primary versus secondary), urinary tract infection, or any diurnal urinary symptoms other than daytime incontinence (P > 0.05 for all). of 10 renal scintigrams, 5 (50%) showed renal cortical defects.

**Conclusions:**

VUR is uncommon in children with MNE and in those with NMNE who do not wet themselves during the day; however, it is a relatively common finding in enuretic children who have daytime incontinence. We recommend VCUG in all enuretic children who have daytime incontinence.

## 1. Background

Nocturnal enuresis often accompanies urological abnormalities (1), and urinary incontinence can be caused by anatomic or neurologic abnormalities, including vesicoureteral reflux (VUR), ectopic ureter, bladder exstrophy, myelomeningocele (2), congenital urethral stricture (3), anterior urethral valve (4), and PUV (5). The prevalence of VUR is higher in enuretic children (6-8) than in other children (9, 10). New studies have noted VUR in 6–23% of children with enuresis (1, 11, 12), while other urological abnormalities have been reported in a few cases (1, 11, 12).

The American Academy of Pediatrics recommends urological evaluation of enuretic children only in cases with a history of urinary tract infection (UTI) (13), while other studies suggest such investigations in children with non-monosymptomatic nocturnal enuresis (NMNE) (14), enuretic children with bladder irritability symptoms (urgency or frequency) (15), children with secondary enuresis (16), and children who have failed conventional medical therapy (17). In addition, a recent study recommended VCUG for severe nocturnal enuresis (patients who wet every night) (11).

Renal and upper collecting system abnormalities and urethral outflow obstruction are rarely found in enuretic children; therefore, routine cystoscopy or intravenous pyelography (IVP) is not recommended (1, 17). The association between daytime incontinence, uninhibited bladder contraction, and VUR is well established (18-20). Elimination disorders are VUR-associated factors that always worsen the prognosis and increase the risk of UTI and upper tract damage (21).

## 2. Objectives

This study was conducted to identify children with nocturnal enuresis who are at greater risk for lower urinary tract urological abnormalities.

## 3. Patients and Methods

Neurologically normal children who were referred to the nephrology clinic of Dr Sheikh Children Hospital during the 3-year period of 2007–2009 with a chief complaint of nocturnal enuresis were prospectively evaluated. Mentally retarded patients and those with neurological abnormalities (cerebral palsy, myelodysplasia, spinal injury or surgery) and patients who did not consent to do VCUG were excluded from the study. Enuresis, its subtypes, and lower urinary tract terminology were defined according to the International Children’s Continence Society (ICCS) criteria (22). Urine analysis (U/A), urine culture (U/C), and kidney and bladder ultrasonography (US) were obtained in all patients. Bladder US was used to estimate bladder volume (BV), bladder wall thickness (BWT), and post void residual urine (PVRU). BWT > 3 mm in a full bladder and PVURV ≥ 15cc were considered abnormal and BV was compared to the normal range for age (23). Urodynamic studies (UDS), including uroflowmetry, pelvic floor electromyography (EMG), and cystometrography (CMG), were done in 58 patients who participated in a research study funded by a research grant from Mashhad University of Medical Sciences. This portion of the study was approved by the local ethics committee, and oral consent was obtained from the children or their parents. Oral consent was also obtained for VCUG.

Urological evaluation was performed by VCUG. Inclusion criteria for urological evaluation included abnormal US, daytime urinary incontinence, abnormal UDS, UTI, or a history of VUR in their siblings as measured by VCUG. A Tc-99m-DMSA scan was obtained in cases of VUR or abnormal kidney US findings suggesting renal parenchymal injury (decreased renal size, renal scarring, and decreased renal cortical thickness).

For data analysis, Chi square, Fisher’s exact, and the Student’s t-tests were used, and a P value of <0.05 was considered statistically significant.

## 4. Results

A total of 115 children were referred, and 60 children (26 boys and 34 girls) aged 5–17 (mean ± SD: 8.46 ± 2.45) years met the inclusion criteria and were enrolled in the study. Twenty-eight (46.7%) children had MNE and 32 (53.3%) children had daytime symptoms and categorized as NMNE. Enuresis was primary in 50 (83.3%) children and secondary in 9 (15%) children. The parents of 1 child were not sure about the primary or secondary nature of disease. Thirty-seven children (61.7%) had a family history of enuresis in their close relatives, whereas the remaining 11 (18.3%) had no family history. Parents of 12 (20%) children were not sure about their family history. The number of bedwettings was 1–7 (mean ± 2: 5.4 ± 2) night per week.

VUR and posterior urethral valve (PUV) were reported in 10 (16.7%) patients and 1 (1.7 %) patient, respectively. VUR was reported in 10 patients and 12 kidney ureter units (KUU) .The grades of VUR were I (1), III (7), IV (2), and V (2), According to results of VCUG, patients were divided into 2 groups, VUR positive (VUR^+^) and VUR negative (VUR-). Table 1 shows a comparison of the US and VCUG results in the VUR^+^ and VUR^-^ groups.

**Table 1:** US and VCUG Findings in the VUR^+ ^and VUR^ -^Groups

US ^a^ Bladder Findings			
	All Patients, No. (%)	VUR^+^^a^ Group, No. (%)	VUR^-^ Group, No. (%)
Normal	7 (100)	0 (0)	7 (100)
Increased bladder wall thickness	43 (100)	8 (18.6%)	35 (81.4)
Irregularity of bladder wall	37 (100)	5 (13.5)	32 (86.5)
Post void urinary residue	14 (100)	3 (21.4)	11 (78.6)
Increased bladder volume	1 (100)	0 (0)	1 (100)
Widening of bladder neck	2 (100)	1 (50)	1 (50)
Decreased bladder volume	2 (100)	0 (0)	2 (100)
Total number	60 (100)	10 (16.7)	50 (83.3)
	**VCUG^a^Findings (Other Than VUR)**		
**VCUG** **(other than VUR)**	**All Patients,** **No. (%)**	**VUR^+^ Group** **No. (%)**	**VUR^-^Group** **No. (%)**
Normal	36 (100)	7 (19.5)	29 (80.5)
Irregularity of bladder wall	14 (100)	3 (21.4)	11 (78.6)
Vertical bladder	5 (100)	1 (20)	4 (80)
Widening of bladder neck	8 (100)	0 (0)	8 (100)
Spinning top deformity	2 (100)	0 (0)	2 (100)
Enlarged bladder	2 (100)	1 (50)	1 (50)
PUV^a^	1 (100)	0 (0)	1 (100)
Bladder wall diverticulum	3 (100)	1 (33.3)	2 (66.6)
Total	60 (100)	10 (16.7)	50 (83.3)

^a^Abbreviations: PUV, posterior urethral valve; US, ultrasonography; VCUG, voiding cystourethrography; VUR, vesicoureteral reflux

Statistical analyses were performed to determine the differences in clinical data between the VUR^+^ and VUR^-^ groups (Table 2). Interestingly, all cases of VUR were found in patients with primary enuresis. VUR was significantly more frequent in children with daytime incontinence and enuretic girls (P = 0.016 and 0.003, respectively). It was remarkable that 4 (40%) children with nocturnal enuresis and VUR had a family history of enuresis (Table 2).

**Table 2:** Clinical Findings in VUR^+^^a^ Children and VUR^-^ Children with Enuresis

Clinical Parameter	Patients No. (%)	VUR^+^^a^, No. (%)	VUR^-^, No. (%)	*P*-Value Measure- ment
Age				1
≤10 y	46(100)	8(17.4)	38(82.6)	
>10 y	14(100)	2(14.3)	12(85.7)	
Gender				0.003
Female	34(100)	10(29.4)	24(70.6)	
Male	26(100%)	0(0)	26(100)	
Positive family history ^b^	37(100)	4(10.8)	33(89.2)	0.609
Negative family history	11(100)	2(18.2 )	9(81.8)	
Daytime incontinence	32(100)	9(28.2)	23(71.8)	0.016
Daytime continence	28 (100)	1(3.6 )	27 (96.4 )	
Abnormal defecation	15(100)	4(26.7)	11(73.3 )	0.25
Normal defecation	45(100)	6(13.3)	39(86.7)	
Positive history of UTI	22(100)	5(22.7)	17(77.3)	0.494
Negative history of UTI	36(100)	4(11.1)	32(88.9)	
Primary enuresis ^c^	50(100)	10(20)	40(80)	0.333
Secondary enuresis	9(100)	0(0)	9(100)	
UTI at presentation ^d^	10(100)	4(40)	6(60)	0.055
Sterile urine at presentation	49(100)	6(12.2)	43(87.8)	
Total ^e^	60(100)	10 (16.7)	50 (83.3)	

^a^Abbreviation: VUR, vesicoureteral reflux

^b^In 12 cases, the parents were unsure of their family history.

^c^History of UTI was unclear in 1 patient.

^d^In 1 case, the parents were not sure if the enuresis was primary or secondary.

^e^Urine culture was not performed in 1 patient.

Nine of 10 (90%) children with VUR had daytime incontinence. The mean ages of the children in the VUR^+^ and VUR^-^ groups were 7.72 ± 1.95 and 8.61 ± 2.5 years, respectively (P = 0.308). Children in the VUR^+^ in VUR^-^ groups wet the bed an average of 6.12 ± 1.64 and 5.44 ± 2.09 nights per week, respectively (P = 0.388).

A 10-year-old boy with NMNE who had minor daytime symptoms and daytime continence was found to have PUV. Interestingly, the child’s father had a history of nocturnal enuresis in childhood. We did not find any correlation between VUR and the form of enuresis (primary or secondary), UTI, or diurnal urinary symptoms, except for daytime incontinence (P > 0.05 for all) (Tables 2, Table 3).

**Table 3:** Urinary and Bowel Symptoms in VUR^ +^^a^ Children and VUR^ - ^Children With Enuresis

Symptom	All Patients, No. (%)	VUR^+^ Group, No. (%)	VUR^-^ Group, No. (%)	*P* Value
Daytime urinary leakage	29(48.3%)	6(60%)	23(46%)	0.5
Incontinence urinary	10 (16.7)	4 (40)	6 (12)	0.052
Increased voiding frequency	10 (16.7)	3 (30)	7 (14)	0.347
Straining	1 (1.7)	0 (0)	1 (2)	1
Dribbling	2 (3.3)	0 (0)	2 (4)	1
Giggle incontinence	2 (3.3)	0 (0)	2 (4)	1
Urge incontinence	12 (20)	3 (30)	9 (18)	0.43
Holding maneuver	7 (11.7)	1 (10)	6 (12)	1
Wetting during nap	2 (3.3)	0 (0)	2 (4)	1
Interrupted urinary stream	2 (3.3)	0 (0)	2 (4)	1
Constipation	8 (13.3)	1 (10)	7 (14)	1
Encopresis	10 (16.7)	4 (40)	6 (12)	0.052
Decreased voiding frequency	3 (5)	0 (0)	3 (6)	1
No daytime urinary or bowel symptom	19 (31.7)	1(10)	18 (36)	0.148
Total	60 (100)	10 (100)	50 (100)	

^a^Abbreviation: VUR, vesicoureteral reflux

In our series, VUR was found to be associated with abnormal bladder elimination (daytime incontinence, decreased or increased bladder frequency) in 9 out of 32 (25.6%) patients who were in the NMNE group, while in children with normal bladder elimination (the 28 patients in the MNE group) VUR was reported in only 1 patient (3.6%) (P > 0.05) (Table 3).

Although bladder elimination symptoms were more common in VUR+ children, the difference was not significant (P > 0.05 for all) (Table 3). We noted that constipation was more common in the VUR- group, while encopresis was more common in VUR+ patients. Five of 18 (27.7%) patients with abnormal bowel symptoms had VUR (Table 3). A Tc- 99m-DMSA scan was done in all cases with VUR, and 5 of 10 (50%) renal scintigrams showed unilateral or bilateral renal cortical defects.

## 5. Discussion

VUR has been reported in enuretic patients with different incidence rates (24, 25). Shinsuke et al. (12) reported VUR in 31 of 135 (23%) of the children with enuresis who underwent VCUG, while Yasuyuki (11) found VUR in86 KUU in 70 (6.4%) of 1088 patients, and Kawauchi (1) noted urological abnormalities on VCUG in 7.1% of 695 enuretic children. Although different studies have suggested that VUR is more common in enuretics than in the normal population, there is no general consensus on the groups of enuretic children who should be evaluated for lower urinary tract abnormalities, especially VUR (13-17).

Different studies have been conducted to define the risk of congenital lower urinary tract anomalies in enuretic children without considering the type of enuresis or their response to treatment (1, 6, 8). They have found VUR in 6.4–16% of patients, an incidence similar to that in our series (16.7%). We selected patients according to the recommendations of previous studies (13-17). Therefore, it should be considered that the overall incidence of VUR in enuretic patients might be lower than that we found.

Our findings are consistent with Kajwara’s study, which showed VUR more frequently in children with NMNE than in children with MNE (26). In contrast to our study, which noted VUR only in cases with primary enuresis, Abrams et al. stated that secondary enuresis is more likely to be associated with an organic cause (27), while the study by Robson et al. noted that the prevalence of VUR did not differ significantly between cases of primary and secondary enuresis (28). Similar to Husman’s study (29), we noted some significant urological abnormalities in a minority of children with primary MNE.

In contrast to the studies by Shinsuke and Yasuyuki (11, 12), which reported low-degree VUR in the majority of affected cases, in our series, the grade of VUR was moderate in most patients and severe in 3 patients.

Nocturnal enuresis can cause renal damage, especially when recurrent UTI or VUR exists (30). Due to the moderately high incidence of renal parenchymal damage in patients with enuresis and UTI, McDermott et al. recommended considering infection as an indication for further investigation (performing VCUG) (25). In our series, 5 of 10 patients with VUR had a UTI. Two patients without history of infection and 3 patients with UTI showed renal cortical damage on renal scintigram. We did not obtain a Tc-99m-DMSA scan for all cases with a history of UTI, and in our series, only a few patients were evaluated for renal damage, so the results showing the correlation between UTI and renal damage are not statistically valid. In contrast to previous studies (14-17), we did not find a correlation between VUR and UTI, secondary enuresis, or any daytime symptoms except for incontinence (P > 0.05 for all) .It is surprising that Nielsen’s study suggested enuresis as a protective factor against nephropathy in patients with VUR (24).

Dysfunctional elimination syndrome (DES), which refers to an abnormal pattern of bowel and bladder elimination with unknown etiology, usually presents in toilet-trained children without underlying anatomic or neurologic abnormalities. It has been reported that VUR and DES are associated (31, 32). Two kinds of elimination disorders are associated with VUR, pure bladder elimination disorder and combined bladder and bowel elimination disorder. DES is always a factor that worsens prognosis of VUR, increase the risk of infections complications and renal damage (33).

An association between bowel and urinary symptoms has been reported, and chronic constipation has been suggested as a risk factor for urological problems (21, 33). Kawauchi et al. (1) suggested that the risk of UTI and urgency is increased in chronic functional constipation; however, only pollakiuria (severely increased voiding frequency) was statistically more frequent in patients with urological abnormalities than in patients without them. In our series, abnormal urinary and bowel elimination symptoms, such as urinary incontinence, increased voiding frequency, urge incontinence, and encopresis were common findings in patients with VUR (P > 0.05 for all), while the majority of patients with constipation did not have urological abnormalities (P > 0.05) (Table 3).

We found renal cortical damage in 5 of 60 (8.3%) enuretic children. This finding differs from the general belief of a favorable course of childhood enuresis. We did not find any significant correlation between a family history of enuresis and the absence of congenital anomalies of the urinary tract (VUR). In fact, 4 (40%) of the children with VUR and the single child with PUV had a family history of enuresis. This finding suggests that a family history of enuresis in close relatives does not guarantee the absence of urological abnormalities.

While VUR is uncommon in children with MNE and those with NMNE who do not wet themselves during the day, it is a significantly common finding in enuretic children with daytime incontinence. Our study suggests that VUR might be more frequent in females. As few studies have evaluated the relation between gender and VUR in enuretic children, we believe that additional studies are needed to prove or disprove this association. We noted that children with NMNE who have daytime incontinence are at increased risk for VUR; thus, VCUG is recommended for this group of enuretic children.
